# Challenges during Colonoscopy in Women with a Prior Hysterectomy

**DOI:** 10.12669/pjms.37.1.3856

**Published:** 2021

**Authors:** Lubna Kamani, Nazish Butt, Hanisha Khemani

**Affiliations:** 1 Lubna Kamani, FCPS, MRCP (UK), FRCP (London), FACG. Associate Professor, Department of Gastroenterology, Liaquat National Hospital, Karachi, Pakistan. Consultant Aga Khan University Hospital, Karachi, Pakistan; 2 Nazish Butt, Assistant Professor, Department of Gastroenterology, Jinnah Post Graduate Medical Centre, Karachi, Pakistan; 3 Hanisha Khemani, Gastroenterology Resident, Department of Gastroenterology, Jinnah Post Graduate Medical Centre, Karachi, Pakistan

**Dear Editor,**

Colonoscopy is a technically difficult procedure due to mobility and tortuous anatomy of the colon. It becomes more challenging in women because of deeper transverse colon and a broad pelvis. This challenge further deepens in patients with prior history of a hysterectomy because of multiple adhesions formation leading to acute angulation of sigmoid colon as well as the bowel dipping deeper into the pelvis.^1^ Apart from standard indications of colonoscopy in females, including bleeding per rectum, recent change in bowel habit, screening for colorectal cancer, unexplained anemia or weight loss, there is sometimes occurrence of alteration in defecation and bowel function post hysterectomy and colonoscopy is often required to rule out organic causes.^2^

The rate of moderate or severe discomfort varies significantly between women who had undergone a hysterectomy and those who had not. Mild discomfort was noticed in women without a hysterectomy and moderate to severe discomfort was noticed in women with a hysterectomy.^3^

A meta-analysis by Clancy et al highlighted that colonoscopy completion rate appeared to be decreased in women with a history of hysterectomy in comparison to women without it.^4^ Additionally post hysterectomy patients not only experience more discomfort during procedure resulting in higher doses of analgesia but also has a higher chance of perforations because of sharp bends and bowel tethering.^1^ In order to address these issues, colonoscopist should opt for more flexible colonoscope which will help them to navigate tight angulations. The pediatric colonoscope has shown to be superior as compared to the adult colonoscope for routine colonoscopy in women with fixed and angulated sigmoid colon after pelvic surgery.^5^ Changing position of the patients to supine or right lateral can lead to alteration of bowel anatomy resulting in facilitation of scope through the angulated segment. Moreover, applying external pressure on sigmoid can help in negotiating and further movement of the scope. If there is formation of long loop in the redundant sigmoid colon, then using the stiffening device together with withdrawal and de-looping will help in forward movement of the scope with reduced chances of complications.

However, despite these maneuvers if endoscopists still find difficulty in negotiating the scope further in lumen together with increased patient discomfort, the procedure should be abandoned and the patient must get referred to a more experienced colonoscopist at a high volume center. Pre-procedure counselling with the patient explaining the above points is imperative.

## Author Contribution:

**LK:** Conceived the idea and did final editing and review of manuscript and approval.

**NB:** Review of literature and revision of manuscript

**HK:** Writing of first draft of manuscript and review of literature
